# Supplementary figures and images for: Lipopolysaccharide-activated macrophages regulate the osteogenic differentiation of bone marrow mesenchymal stem cells through exosomes
Source: PeerJ. 2022 May 13;10:e13442. doi: 10.7717/peerj.13442 (PMC9109694; doi:10.7717/peerj.13442)

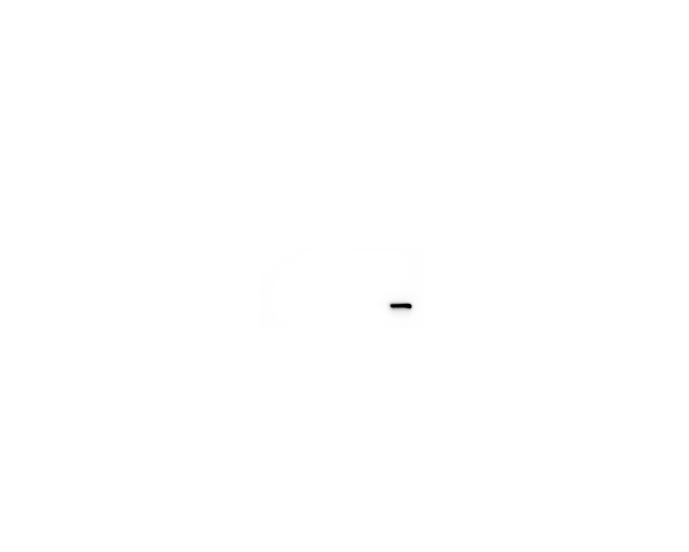

Supplement: Supplemental Information 7 [file peerj-10-13442-s007.zip › calnexin.tif]

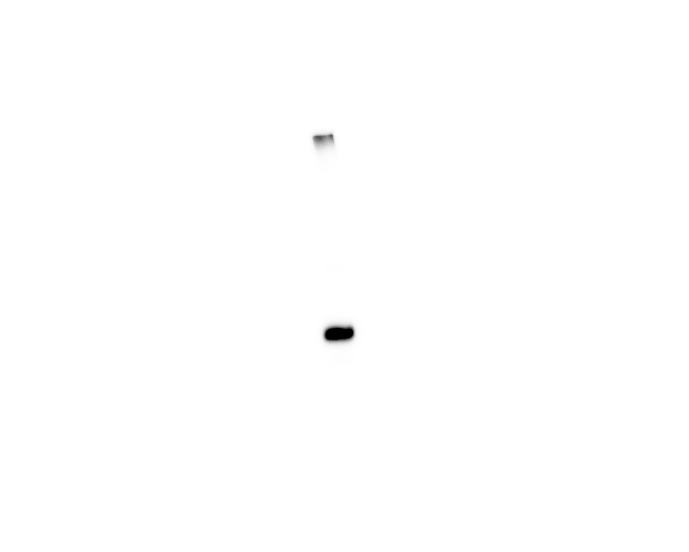

Supplement: Supplemental Information 7 [file peerj-10-13442-s007.zip › CD81.tif]

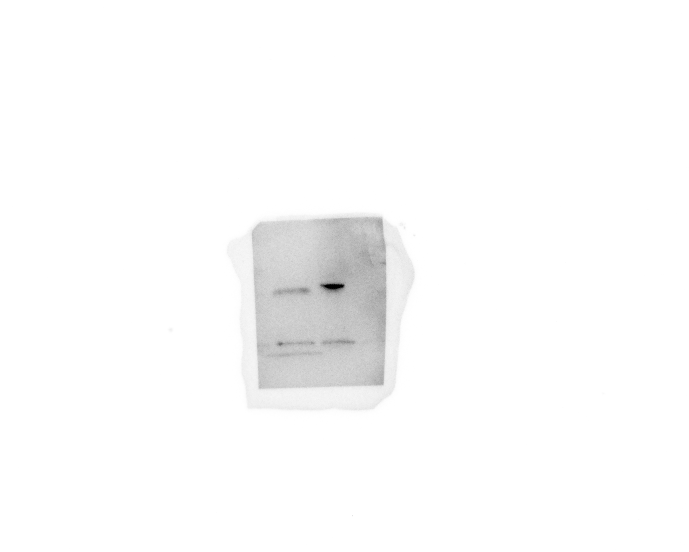

Supplement: Supplemental Information 7 [file peerj-10-13442-s007.zip › CD9.tif]

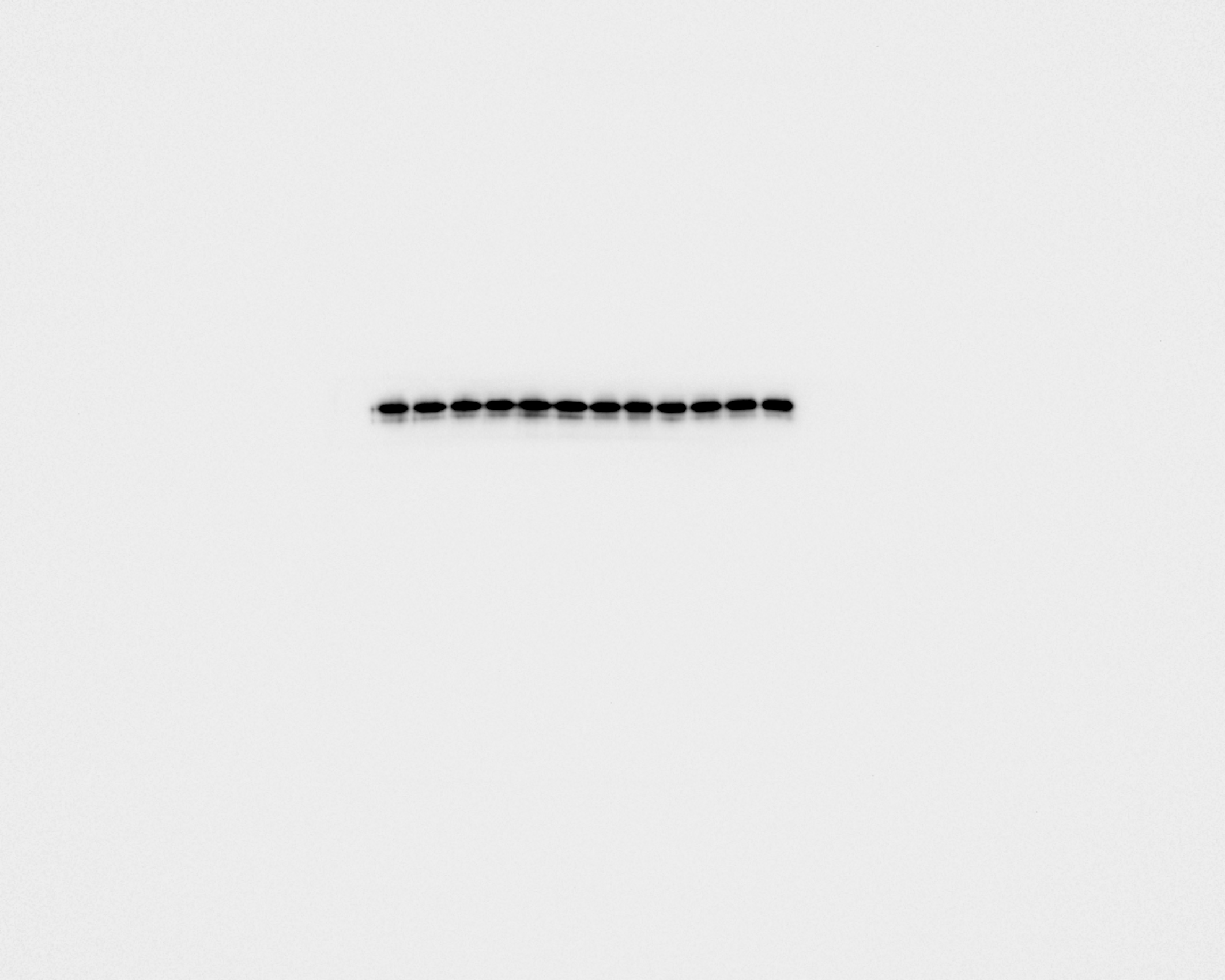

Supplement: Supplemental Information 7 [file peerj-10-13442-s007.zip › GAPDH.tif]

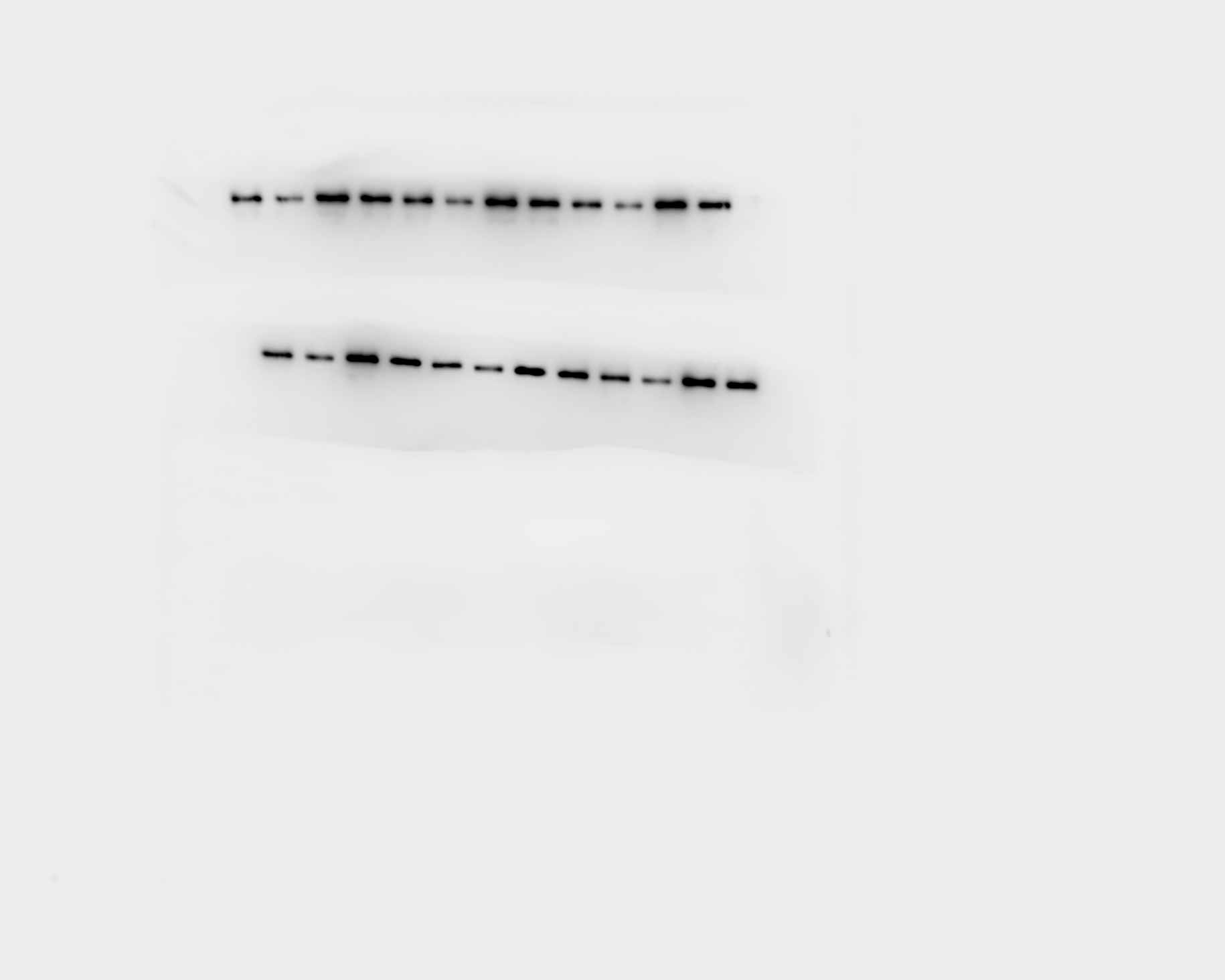

Supplement: Supplemental Information 7 [file peerj-10-13442-s007.zip › RUNX2 COL-1.tif]

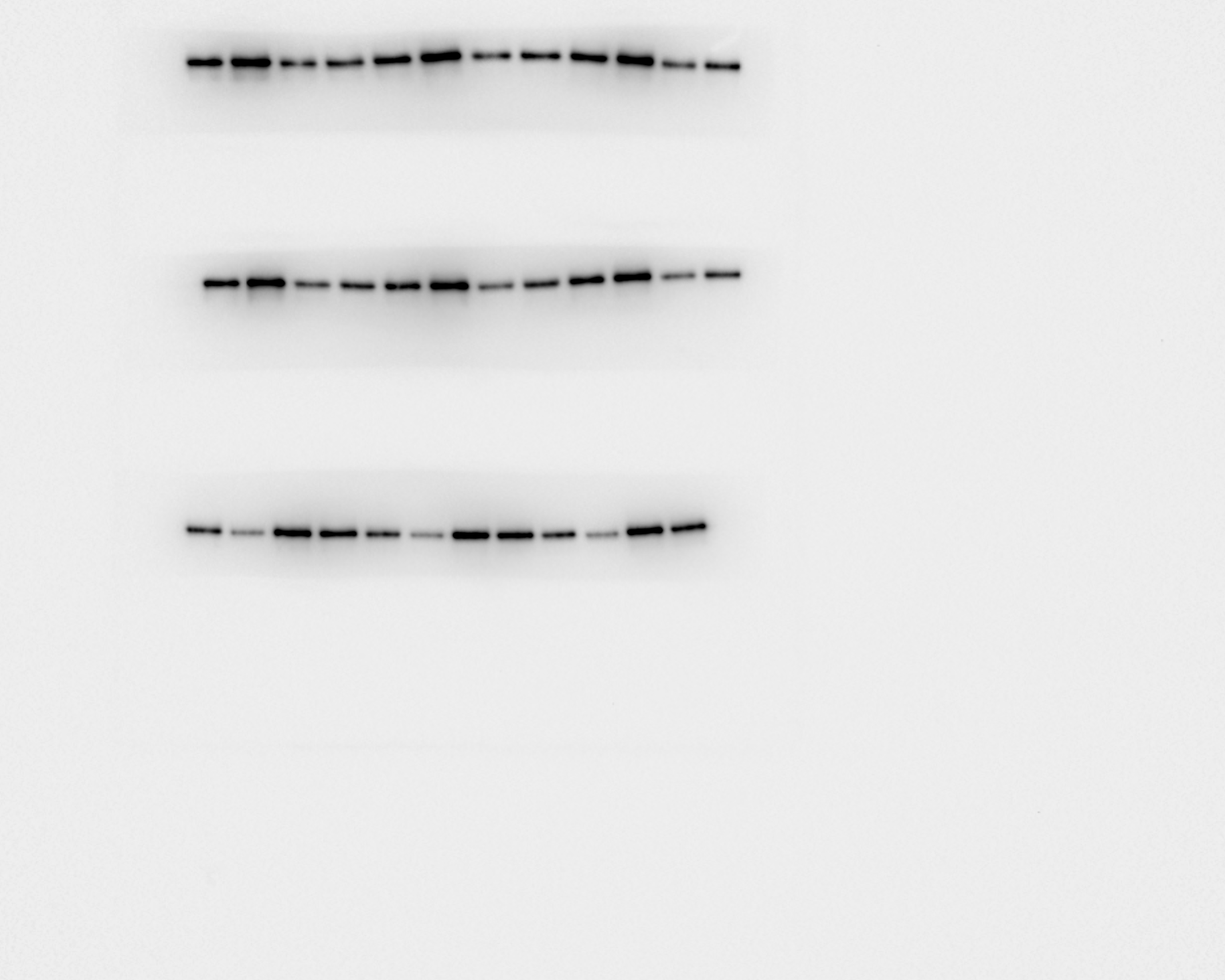

Supplement: Supplemental Information 7 [file peerj-10-13442-s007.zip › TNF-α IL-6 ALP.tif]

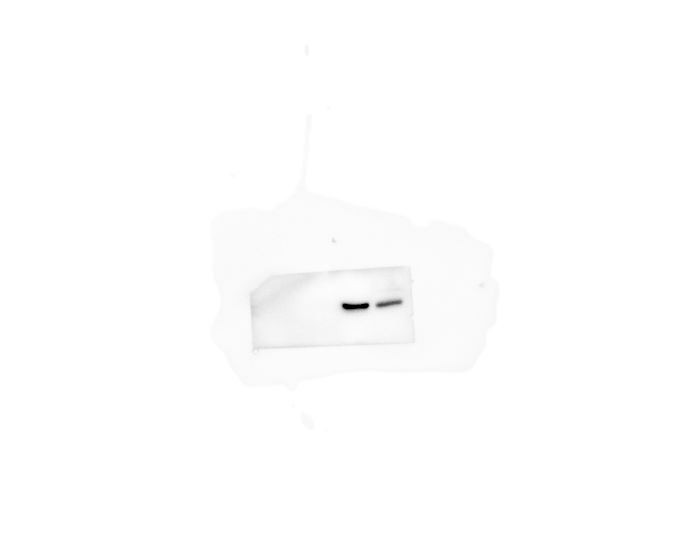

Supplement: Supplemental Information 7 [file peerj-10-13442-s007.zip › TSG101.tif]
